# Artificial Intelligence Reveals Nature: Functional Parallels Between a Designed and a Natural Peptide

**DOI:** 10.3390/ijms262110607

**Published:** 2025-10-31

**Authors:** Jiashu Wang, Thomas David Daniel Kazmirchuk, Maryam Hajikarimlou, Mustafa Al-Gafari, Sarah Takallou, Houman Moteshareie, Frank Dehne, Bahram Samanfar, Mohan Babu, Taha Azad, Ashkan Golshani

**Affiliations:** 1 Ottawa Institute of Systems Biology, Faculty of Medicine, University of Ottawa, Ottawa, ON K1H 8M5, Canada; 2 Department of Biology, Faculty of Science, Carleton University, Ottawa, ON K1S 5B6, Canada; 3 Institute of Biochemistry, Faculty of Science, Carleton University, Ottawa, ON K1S 5B6, Canada; 4 Heathy Environments and Consumer Safety Brance, Health Canada, Ottawa, ON K1A 0K9, Canada; 5 School of Computer Science, Faculty of Science, Carleton University, Ottawa, ON K1S 5B6, Canada; 6 Ottawa Research and Development Centre, Agriculture and Agri-Food Canada, Ottawa, ON K1A 0C6, Canada; 7 Department of Biochemistry, University of Regina, Regina, SK S4S 0A2, Canada; 8 Department of Microbiology and Infectiology, Faculty of Medicine and Health Sciences, Université de Sherbrooke Cancer Research Institute, Université de Sherbrooke, Sherbrooke, QC J1E 4K8, Canada; 9 Centre de Recherche du Centre Hospitalier Universitaire de Sherbrooke, Centre Intégré Universitaire de Santé et de Services Sociaux de l’Estrie–Centre Hospitalier Universitaire de Sherbrooke, Sherbrooke, QC J1H 5N4, Canada

**Keywords:** AI-assisted drug design, theranostic peptides, natural peptides, synthetic peptides, artificial intelligence, SARS-CoV-2

## Abstract

Natural peptides derived from plants have been an important source of medical substances for several decades. Due to their mechanism of action, chemical potential, and favourable side effect profile, these peptides represent a safer alternative to synthetic pharmaceutical treatments. In this study, we report the discovery of a natural peptide derived from the *Brassica napus* (Canola) proteome that exhibits high functional similarity to an artificial intelligence (AI)-generated peptide that is designed to bind to the severe acute respiratory syndrome coronavirus 2 (SARS-CoV-2) spike 1 (S1) protein receptor-binding domain (RBD) region. The results of a series of experiments including molecular docking simulations, as well as binding and inhibition assays suggest that the natural peptide exhibits functions similar to those of the AI-generated peptide in binding to the RBD region and disrupting its interaction with the human host receptor angiotensin-converting enzyme 2 (ACE2). This study demonstrates the potential of AI-designed peptides to facilitate the identification of natural peptides with similar functional properties.

## 1. Introduction

Traditional processes of drug discovery for therapeutics and diagnostics (theranostics) usually take decades to be completed [1,2]. In contrast, the peptide designs driven by artificial intelligence (AI) may significantly reduce the cost and development time [3,4]. Peptide-based theranostics have gained significant attention due to their ability to specifically target proteins with high affinity while also minimizing off-target interactions [4]. Their smaller size enhances tissue penetration compared to larger antibody-based drugs [5]. Additionally, peptides offer lower production costs and eliminate the batch-to-batch variability often observed in antibody manufacturing [6,7,8]. Advances in AI-driven peptide design have enabled the rapid discovery of novel peptides with optimized binding properties [9,10]. AI-guided approaches utilize computational algorithms to generate peptides that selectively bind to a target of interest, making them valuable tools in drug discovery. Their high precision can make these peptides suitable for diagnostic applications [11,12,13] in addition to therapeutics [14]. The recent Coronavirus disease-19 (COVID-19) pandemic spurred the development of many computational approaches for designing anti-severe acute respiratory syndrome—coronavirus-2 (SARS-CoV-2) peptides [15,16,17,18,19].

Natural peptides, particularly those derived from plants, offer unique advantages over synthetic peptides [20,21,22]. These advantages include lower immunogenicity, reduced toxicity, potential evolutionary optimization for stability and bioavailability, and possibly easier regulatory approval (e.g., Food and Drug Agency (FDA) clearance) [8,23,24,25,26,27,28,29,30,31]. Given these benefits, identifying natural peptides with functional properties similar to AI-designed peptides could provide an alternative route to peptide-based therapeutics.

Previously, we utilized a pioneering AI technology [9] to design a series of peptides against severe acute respiratory syndrome coronavirus 2 (SARS-CoV-2) spike (S1) protein receptor-binding domain (RBD) region [16]. This tool employs an algorithm designed to minimize off-target interactions without relying on docking models or requiring information about the target’s 3D structure. It initiates with a diverse pool of randomly generated amino acid sequences. Through multiple generations involving fitness-based selection, mutation, and crossover events, the algorithm mimics natural evolution. It progressively converges on sequences predicted to effectively interact with a specified target while minimizing unintended interactions with non-target proteins [9]. Typically, the optimization process spans 500 to 1000 iterations. These peptides have previously been used for diagnostic purposes [11,16] as well as therapeutics [14]. During our investigations, we identified a naturally occurring peptide called Nat1 from *Brassica napus* (Canola) that shares a high degree of sequence identity with one of our AI-generated peptides called A13 designed to bind the RBD region. This high sequence similarity (88% identity) raises important questions regarding the extent to which AI-generated peptides reflect naturally occurring functional sequences, and whether AI can assist in uncovering bioactive peptides from natural sources.

To address this question, we performed a series of biochemical and functional assays comparing the A13 and Nat1 peptides. Using molecular docking, enzyme-linked immunosorbent assay (ELISA)-based binding assays, an angiotensin-converting enzyme 2 (ACE2)-RBD binding inhibition assay, a *NanoLuc* bioreporter assay, and a pseudovirus infectivity assay, we assessed the ability of both the A13 and Nat1 peptides to bind to the RBD region and prevent the interaction with its human host protein ACE2. Our findings suggest that A13 can have the potential to not only function as therapeutic candidates but also serve as a means of identifying naturally occurring peptides with similar properties, providing a novel approach for peptide-based drug discovery and diagnostics.

## 2. Results

### 2.1. Peptide Design and Molecular Docking Analysis

To evaluate whether A13, the AI-designed peptide, and Nat1, the naturally occurring peptide identified from *Brassica napus* with a high degree of sequence identity to A13, interact in a comparable manner with the SARS-CoV-2 S1 protein, we carried out a series of molecular docking analyses using AlphaFold 3 [32]. Molecular docking is a widely used computational model that aims to predict the most favorable binding conformations between a ligand and its target protein by taking into account structural complementarity as well as the energetic stability of the resulting complex [33,34]. This technique can allow researchers to gain certain mechanistic insights into how potential therapeutic molecules may engage their biological targets prior to extensive experimental testing.

**Figure 1:**
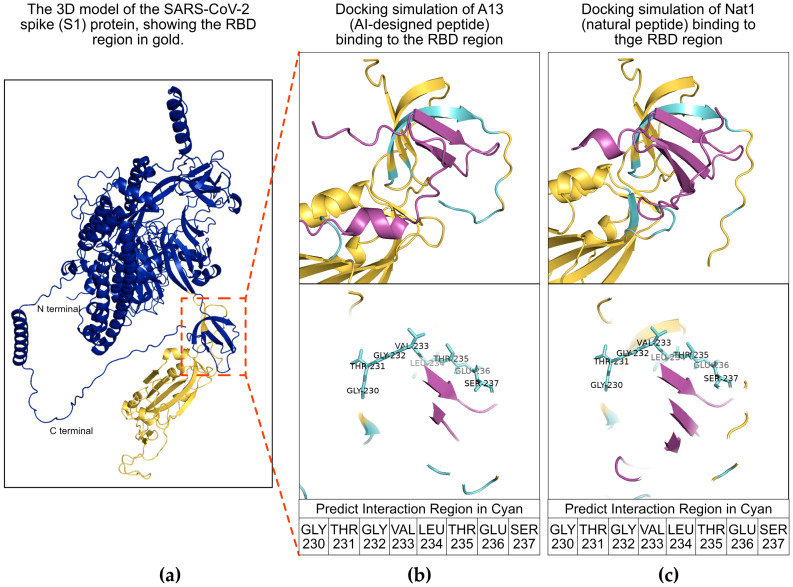
Molecular docking analysis of peptides with the severe acute respiratory syndrome coronavirus 2 (SARS-CoV-2) spike 1 (S1) protein receptor-binding domain (RBD) region. (**a**) Schematic illustration of the viral S1 protein. The S1 protein is shown in dark blue and its RBD region is shown in gold. The red inset box highlights the predicted interaction region between the RBD region and the peptides. (**b**,**c**) Docking simulations of A13 and Nat1 binding to the RBD region, respectively. The RBD is shown in gold, peptides are shown in purple, and interacting regions are shown in cyan. Amino acid residues of the RBD involved in peptide interactions are indicated as GLY230, THR231, GLY232, VAL233, LEU234, THR235, GLU236 and SER237.

The docking simulations performed here consistently indicated that both A13 and Nat1 were able to associate with the RBD of the spike protein and that the interaction profiles were highly similar between the two peptides (Figure 1). The RBD is a critical subregion of the S1 protein and is well established as the site that directly contacts the host receptor ACE2, thereby mediating viral attachment and entry into human cells [35]. The predicted dockings revealed overlapping sets of contact residues within the RBD, particularly in a portion of the domain that is located in close proximity to the receptor-binding motif responsible for ACE2 recognition. Specifically, the residues GLY230, THR231, GLY232, VAL233, LEU234, THR235, GLU236, and SER237 were identified as forming the main interface with both peptides, suggesting that these amino acids may play a role in stabilizing the interaction. These contact points, which are highlighted in Figure 1 panels (b) and (c), imply that A13 and Nat1 may engage the S1 protein in a very similar fashion. Taken together, these predictions provide an initial support for the hypothesis that both the engineered and natural peptides may share a common mechanism of activity, potentially interfering with the RBD–ACE2 interaction that is essential for viral entry.

### 2.2. Target Binding Analysis

**Figure 2:**
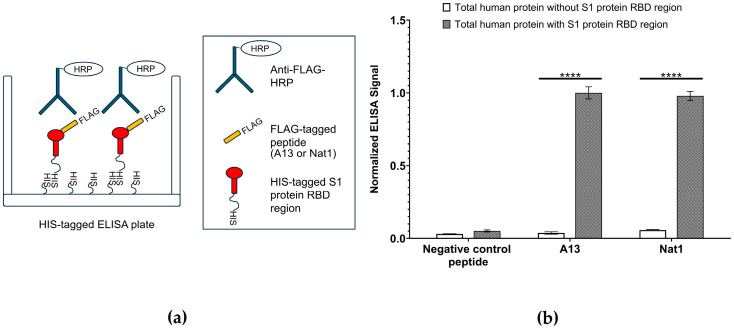
Enzyme-linked immunosorbent assay (ELISA)-based target binding assay. (**a**) Schematic illustration of ELISA experiment for testing the binding A13 and Nat1 to the viral RBD region. ELISA was performed using immobilized HIS-tagged SARS-CoV-2 S1 protein RBD region and FLAG-tagged A13 or Nat1 with or without total human protein (25 ng/mL). Binding was detected using an anti-FLAG antibody conjugated to HRP. (**b**) Binding of A13 and Nat1 peptides to RBD. The signal value of A13 with human total protein was set as 1.00 and others were normalized to this number. Data represent the mean value from at least three independent experiments. Error bars represent standard deviation. Statistical significance was determined using the nonparametric two-tailed Student’s *t*-test (**** *p* ≤ 0.0001).

Next, to assess the binding affinity of the designed peptides toward the S1 protein RBD region, an ELISA-based binding assay was performed (Figure 2). ELISA is a widely used, plate-based biochemical assay that enables the detection and quantification of molecular interactions through antibody-mediated recognition and enzymatic signal development. In this case, the assay was adapted to detect peptide–protein binding events. Briefly, ELISA plates were coated with a solution containing 25 ng/mL of total human protein. To distinguish specific interactions, two coating conditions were prepared: one supplemented with recombinant HIS-tagged S1 protein RBD region and one lacking the recombinant protein. Following immobilization and washing, wells were incubated with FLAG-tagged versions of the A13 and Nat1 peptides, allowing them to interact with either the RBD-containing or RBD-free protein matrix. Bound peptides were then detected using a horseradish peroxidase (HRP)-conjugated anti-FLAG monoclonal antibody, which generates a quantifiable colorimetric output upon addition of a chromogenic substrate.

The results of the ELISA analysis demonstrated that both A13 and Nat1 peptides showed noticeable binding to the immobilized RBD region, while minimal signal was observed in the absence of RBD, indicating background-level binding only. Indicated in Figure 2, binding values were normalized by setting the signal for A13 peptide against total human protein containing the S1 protein RBD region to 1.00. The presence of RBD produced a significant signal relative to the condition without RBD. A similar pattern was observed for Nat1 peptide, which also exhibited a significant increase in binding when RBD was present. By contrast, a peptide carrying a random amino acid sequence and used as a negative control displayed only negligible binding to both RBD-containing and RBD-lacking protein mixtures that were similar to the background level. Statistical significance for all comparisons was determined using the nonparametric two-tailed Student’s *t*-test.

Overall, these findings indicate that both A13 and Nat1 peptides are capable of specifically binding to the S1 protein RBD region with comparable signal intensities, and that their interactions are dependent on the presence of the S1 protein. Importantly, background level of measurable binding in the negative control further validates the assay design and underscores the selectivity of the peptides against the RBD. These results suggest that the two peptides demonstrate similar apparent binding to the S1 protein RBD, supporting their potential utility as molecular tools for detecting or diagnosing with RBD-mediated interactions.

### 2.3. Interaction Inhibition Analysis Between RBD and ACE2

In the ELISA experiments described above, both A13 and Nat1 peptides exhibited high binding recognition for the RBD region of the S1 protein while showing no significant interaction with unrelated human proteins, suggesting a high degree of specificity. In general, the introduction of novel peptides that target a particular protein domain may interfere not only with direct binding but also with interactions between that protein and its physiological binding partners. This possibility raised the question of whether the peptides could also disrupt natural host–virus recognition pathways mediated by the RBD. Based on this, we hypothesized that peptide A13 and Nat1, in addition to demonstrating strong affinity against the RBD region, to similar degrees might act as inhibitors of RBD-host interactions by competitively occupying the same binding interface. Among the known partners, human ACE2 is well established as the primary cellular receptor that recognizes the RBD region of the S1 protein and is required for viral entry during infection [35]. Thus, ACE2 represents the most physiologically relevant candidate for assessing the inhibitory potential of the peptides.

**Figure 3:**
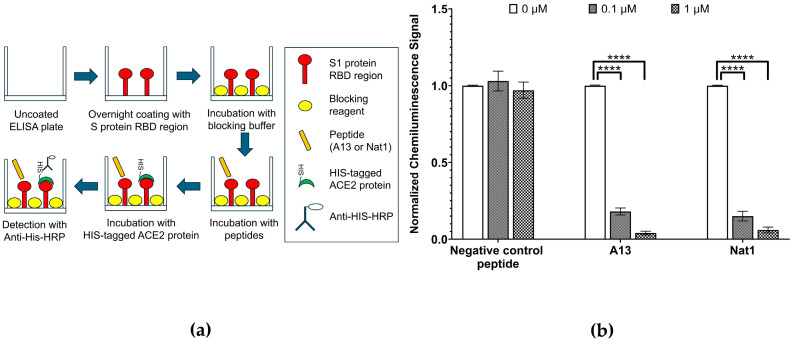
RBD-ACE2 interaction inhibition assays. (**a**) Schematic illustration of ELISA experiment to evaluate the ability of the A13 or Nat1 to prevent the interaction of SARS-CoV-2 S1 protein RBD to human receptor ACE2. First the plates were incubated with immobilized RBD. They were then inoculated with peptides prior to the addition of recombinant human receptor ACE2. (**b**) Inhibition of RBD-ACE2 binding via A13 or Nat1. The level of RBD-ACE2 binding was measured by chemiluminescence. The negative control with no peptide representing RBD-ACE2 interaction was set to 1.00 (3.2 × 10^4^ RLU) and other values were normalized to this number. To the same extent, at the concentration of 0.1 µg/mL, both peptides reduced ACE2 binding compared to the control. At 1.0 µg/mL, inhibition was further enhanced. The negative control peptide did not exhibit a notable inhibition. Data represent the mean value from at least three independent experiments. Error bars represent standard deviation. Statistical significance was determined using the nonparametric two-tailed Student’s *t*-test (**** *p* ≤ 0.0001).

To investigate this possibility, we employed an ELISA-based RBD–ACE2 binding inhibition assay (Figure 3). This assay was designed in a competitive binding format, in which the peptides were first incubated with immobilized S1 protein RBD before the addition of recombinant ACE2 protein carrying a HIS tag. In this system, the degree of ACE2 binding is inversely related to the effectiveness of the peptide competitors: stronger peptide binding to RBD should reduce available binding sites for ACE2, resulting in a lower chemiluminescence signal upon detection. Chemiluminescence values therefore can be obtained as a quantitative result for the extent of RBD-ACE2 interaction remaining after peptide treatment. To ensure comparability across conditions, the signal obtained in the absence of peptide (0 µM) was set to 1.00, representing maximal RBD-ACE2 binding, and all other measurements were normalized to this reference value.

The results revealed a clear concentration-dependent inhibitory effect of both peptides on the RBD-ACE2 interaction. At a low peptide concentration of 0.1 µg/mL, ACE2 binding was suppressed to 0.18 ± 0.02 (*p* ≤ 4.52 × 10^−7^) for A13 and 0.15 ± 0.03 (*p* ≤ 1.32 × 10^−6^) for Nat1, corresponding to an 80–85% reduction in signal relative to the no-peptide control. Increasing the peptide concentration to 1 µg/mL resulted in even stronger inhibition, with ACE2 binding reduced nearly to baseline levels: 0.04 ± 0.01 (*p* ≤ 2.51 × 10^−8^) for A13 and 0.06 ± 0.02 (*p* ≤ 1.18 × 10^−7^) for Nat1. Importantly, a negative control peptide consisting of a random amino acid sequence did not show any inhibitory effect at the same concentrations, confirming that the observed reductions were sequence-specific and not attributable to nonspecific binding or assay artifacts. Statistical significance for all comparisons was determined using the nonparametric two-tailed Student’s *t*-test.

Collectively, these findings provide strong evidence that both A13 and Nat1 are able to block the interaction between the RBD region of the S1 protein and its host receptor ACE2 in vitro to a similar extent. While the two peptides displayed slightly different quantitative inhibition values, their overall effects were comparable, as both achieved near-complete suppression of ACE2 binding at higher concentrations. Together, these results demonstrate that A13 and Nat1 not only strongly bind to the RBD region but also show functional inhibitory activity against the RBD-ACE2 interaction. This suggests their potential utility as candidate molecules for disrupting viral entry mechanisms.

### 2.4. Inhibition Analysis Using a NanoLuc Bioreporter Assay

**Figure 4:**
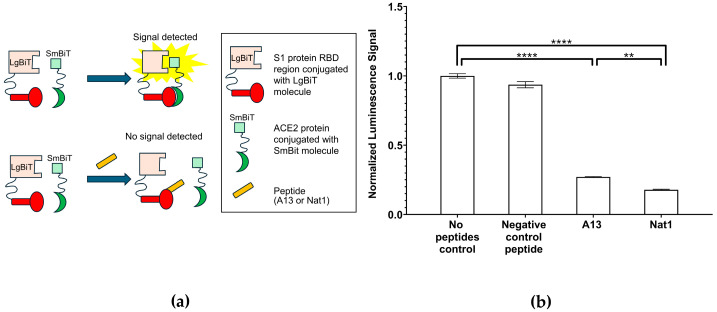
*NanoLuc* bioreporter assays. (**a**) A schematic illustration for the *NanoLuc* bioreporter assay to determine SARS-CoV-2 S1 RBD—ACE2 inhibition by A13 or Nat1. (**b**) *NanoLuc* bioreporter assay indicates that both peptides reduce luciferase complementation to the same extent. The signal value for no peptide control was set to 1.00 (6.30 × 10^5^ RLU) and all other values were normalized to it. In cell lysates, A13 and Nat1 both reduced luminescence, indicating inhibition of RBD-ACE2 binding. The negative control peptide had no significant effect. Data represent the mean value from at least three independent experiments. Error bars represent standard deviation. Statistical significance was determined using the nonparametric two-tailed Student’s *t*-test (**** *p* ≤ 0.0001, ** *p* ≤ 0.01).

To further validate the inhibitory capacity of the peptides on the interaction between the S1 protein RBD region and ACE2, we employed a cell-based *NanoLuc* bioreporter assay (Figure 4). This system provides a sensitive and quantitative method to detect protein–protein interactions within a cellular environment, thereby offering a complementary approach to the plate-based ELISA inhibition assays described earlier. The principle of this assay is based on the reconstitution of *NanoLuc* luciferase from two inactive split fragments: a large fragment (LgBiT) and a small fragment (SmBiT). In the experimental design, the RBD of the S1 protein was fused to the LgBiT domain at the gene level, while ACE2 was fused to the SmBiT fragment [36]. When RBD and ACE2 interact in cells, the two luciferase fragments are brought into close spatial proximity, enabling reassembly of the functional enzyme and leading to a measurable and comparable luminescent signal. Conversely, if peptide inhibitors successfully block the RBD-ACE2 interaction, the luciferase fragments remain separated, the enzyme fails to reconstitute, and the resulting luminescence is diminished.

To enable direct comparison across conditions, the luminescence signal obtained in the no-peptide control group was set to 1.00, and all other measurements were normalized to this value. The results of the *NanoLuc* assay revealed significant inhibition of RBD-ACE2 binding by both A13 and Nat1 peptides. In the presence of the A13 peptide, the normalized luminescence signal in cell lysates decreased to approximately 27% ± 0.3% of the control (*p* ≤ 1.61 × 10^−7^), indicating substantial disruption of the interaction. Similarly, the Nat1 peptide reduced the signal even further, to approximately 18% ± 0.4% of the control (*p* ≤ 1.04 × 10^−7^). Interestingly, when directly comparing the two peptides, Nat1 appeared to exhibit enhanced inhibitory capacity, with an approximately 30% stronger reduction relative to A13 under equivalent experimental conditions. This difference may reflect variations in binding or spatial confirmation interference with the RBD-ACE2 interface. By contrast, the negative control peptide, consisting of a random amino acid sequence, did not cause significant inhibition, producing a luminescence signal of 94% ± 2% of the control, thereby validating the specificity of the effects observed with A13 and Nat1 peptides. Statistical significance across all experimental groups was determined using the nonparametric two-tailed Student’s *t*-test.

All together, these findings provide comprehensive evidence that in the current assay, both peptides can effectively interfere with the RBD-ACE2 interaction, complementing the results obtained with the in vitro plate-based ELISA inhibition assay. Although Nat1 demonstrated stronger inhibition than A13, both peptides showed comparable overall functionality in binding to the RBD and disrupting its interaction with ACE2. These results highlight the potential of the designed peptides to act as competitive inhibitors of the RBD–ACE2 interaction, a critical step in viral entry.

While the cell-based *NanoLuc* bioreporter assay supports the observed inhibitory effects of A13 and Nat1 that were observed in vitro, it is important to acknowledge their limitations. In vitro and cell-based assays do not always fully recapitulate the complexity of in vivo systems, where molecular affinities, receptor densities, and local cellular environments can substantially influence binding dynamics. Therefore, although the observed reductions in luminescence strongly suggest inhibition of RBD-ACE2 binding, additional functional studies are required to determine whether these peptides can effectively block viral entry under more physiological conditions. To address this, we next performed a pseudovirus infectivity assay, which allows direct measurement of viral entry efficiency in the presence or absence of the peptides.

### 2.5. Pseudovirus Infectivity Assay Analysis

**Figure 5:**
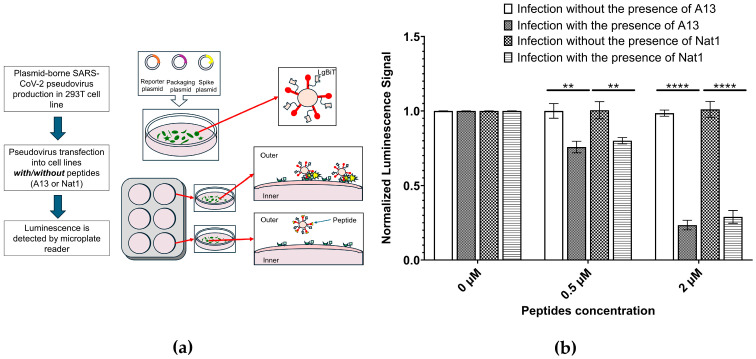
Pseudovirus infectivity assays. (**a**) A schematic illustration for pseudovirus infectivity assay to determine the inhibition ability of the peptides to prevent psuedovirus to enter the cell. (**b**) Pseudovirus infectivity assays indicates that both peptides exhibit similar effects on pseudovirus infected cells. The signal value for pseudovirus infection with no peptide was set to 1.00 and corresponding values were normalized to it. Cells were incubated with pseudovirus in the absence/presence of A13 or Nat1, and infection was measured using a luciferase reporter. Both peptides reduce pseudovirus entry into ACE2-expressing cells. Neither peptide shows any visible effects on the non-peptide sample. Data represents the mean value from at least three independent experiments. Error bars represent standard deviation. Statistical significance was determined using the nonparametric two-tailed Student’s *t*-test (**** *p* ≤ 0.0001, ** *p* ≤ 0.01).

As mentioned above, to better assess whether the peptides could inhibit viral entry into host cells, a lentiviral-based pseudovirus infectivity assay was conducted (Figure 5). This system provides a safe biologically relevant model to study the early stages of SARS-CoV-2 entry, since the pseudoviruses are engineered to present the viral S1 protein on their surface but lack the genetic capacity for replication [37]. Hence, they mimic the contact and entry process of SARS-CoV-2 without posing infectious risk, allowing careful quantitative evaluation of inhibitory compounds under controlled laboratory conditions.

In this assay, cells expressing the ACE2 receptor were incubated with pseudovirus particles in the presence or absence of the test peptides. Successful viral entry was quantified using a luciferase reporter gene encoded within the pseudoviral genome. Once the pseudovirus enters the cell, the luciferase gene is expressed, and its enzymatic activity generates a luminescent signal proportional to the extent of infection. This readout provides a sensitive and quantitative measure of viral entry efficiency, using a mechanism conceptually similar to the *NanoLuc* assay described earlier but reflecting a more physiologically relevant context of viral uptake by host cells. To normalize results across experiments, the luminescence signal for the A13 no-peptide condition (0 µM, no pseudovirus) was set to 1.00, and all other values were expressed relative to this baseline.

The data showed that in the absence of peptide inhibitors, pseudovirus entry proceeded efficiently, with no significant decrease in luminescence observed in the peptide-free control (0 µM). By contrast, both A13 and Nat1 peptides produced measurable and concentration-dependent reductions in pseudovirus infectivity. At 0.5 µM, luminescence signals decreased to 76% ± 3.9% (*p* ≤ 2.63 × 10^−3^) for A13 and 80% ± 2.1% (*p* ≤ 4.45 × 10^−3^) for Nat1, relative to their respective no-peptide controls. These reductions correspond to 20–25% inhibition at low concentrations, suggesting that both peptides are capable of partially blocking viral entry under these conditions. It also suggests that the peptides have potential to further suppress the pseudovirus infection in higher concentrations.

At higher concentrations, inhibition became much more distinct. When the peptide concentration increased to 2.0 µM, viral entry was strongly suppressed, with luminescence signals reduced to 24% ± 3.2% (*p* ≤ 4.40 × 10^−6^) for A13 and 29% ± 4.3% (*p* ≤ 5.37 × 10^−5^) for Nat1, compared to its corresponding control. These values correspond to approximately 70–75% reduction in infectivity, demonstrating that both peptides can potently block pseudovirus entry into ACE2-expressing cells in a dose-dependent manner. Notably, the inhibitory effects of A13 and Nat1 were of similar magnitude at both concentrations tested, indicating that the two peptides share comparable functional capacity despite minor differences in their quantitative profiles. Statistical significance for all comparisons was determined using the nonparametric two-tailed Student’s *t*-test.

An important control in such experiments is to evaluate whether reduced luminescence results from nonspecific cytotoxic effects rather than true inhibition of viral entry. To address this, cells were incubated with peptides in the absence of pseudovirus. Luminescence signals under these peptide-only conditions were indistinguishable from the no-peptide control, confirming that neither A13 nor Nat1 exhibited detectable cytotoxicity at the tested concentrations. This supports the conclusion that the observed decreases in luminescence reflect inhibition of pseudovirus entry rather than peptide-induced impairment of cell viability or reporter expression.

In summary, the pseudovirus infectivity assay demonstrated that both A13 and Nat1 peptides substantially inhibited viral entry into ACE2-expressing cells in a concentration-dependent manner, while showing no signs of cytotoxicity. These results are consistent with the outcomes of the ELISA and *NanoLuc* assays, further supporting that A13 and Nat1 can specifically bind to the RBD region and interfere with its interaction with ACE2. The reason why the inhibitory ability of A13 was stronger than that of Nat1 in vitro, but weaker in vivo, remains unknown. This discrepancy may arise from differences between in vitro and in vivo experimental conditions. One possibility is that, in vivo, an as-yet-undiscovered factor may affect A13 and Nat1 differently Another possibility is that Nat1 may possess a longer intracellular half-life compared to A13.

## 3. Discussion

Our findings demonstrate that AI-designed peptides can serve as tools for identifying functional natural peptides. In the current study, the AI-generated peptide A13 and Canola-derived peptide Nat1 exhibited highly similar binding and inhibitory properties, as shown through biochemical and functional assays. This suggests that AI-based peptide discovery methods may not only generate novel synthetic candidates but may also uncover naturally occurring bioactive molecules with therapeutic potential. The ability of both peptides to effectively bind the RBD region and block its interaction with ACE2 highlights the broader applicability of AI-designed peptides beyond their intended targets. The high sequence similarity observed between the AI-designed peptide and the natural (Canola) peptide raises intriguing questions about evolutionary convergence and the existence of undiscovered natural peptides with antiviral properties.

Natural peptides may offer distinct advantages over synthetic counterparts, including lower immunogenicity, greater stability, and potentially faster regulatory approval [23,24,25,26,27,28,29,30,31]. Moreover, Canola is an edible crop widely consumed globally, which may enhance the perceived safety of its derived peptides. This could be particularly appealing to consumers who, for various reasons, prefer naturally sourced therapeutics over synthetic pharmaceuticals. The familiarity and established safety profile of Canola-derived products (38) may further facilitate acceptance and regulatory approval of such peptides.

By demonstrating that AI-driven approaches can lead to the discovery of functionally relevant natural peptides, this study underscores the value of integrating computational methods with traditional biochemical screening. Future work may focus on expanding AI-guided searches for natural peptides with therapeutic relevance, investigating their mechanisms of action, and assessing their stability and bioavailability. Additionally, structural and evolutionary analyses may provide further insights into the functional relevance of these peptides and their potential applications in antiviral therapeutics. These results suggest that AI-driven peptide discovery may have the potential to bridge the gap between computational design and natural peptide identification, offering new avenues for drug development and diagnostics.

## 4. Materials and Methods

### 4.1. Peptide Design and Molecular Docking Analysis

The AI-designed peptide (A13) was generated using eLIFE also known as as In Silico *Protein Synthesizer (InSiPS)* [9]. In brief, to identify potential peptide candidates, random sequences of approximately 40 amino acids were first generated. Their interaction potential with the target protein was assessed using reference profile of both positive and negative protein–protein interactions (PPIs) databases. Each peptide was then assigned a likelihood score reflecting its predicted binding to the target. To improve these candidates, an iterative optimization process consisting of more than 1000 cycles was applied. In each cycle, individual amino acids within the sequence were systematically modified. If a substitution enhanced the overall likelihood score, the change was retained and carried forward to the next cycle; otherwise, the original residue was preserved. Through this refinement process, peptides with progressively higher predicted binding potential were obtained. Finally, the top-scoring sequences were selected for chemical synthesis and subsequent experimental testing to validate their binding affinity and biological functionality. Within the algorithm, the ability of the peptides to bind to the target is measured using the Protein Interaction Prediction Engine (PIPE) [38]. Sequence homology search was performed using BlastP “https://blast.ncbi.nlm.nih.gov/Blast.cgi?PAGE=Proteins” (accessed on 7 November 2024). Molecular docking was performed using AlphaFold3 [32]. The amino acid sequence of the SARS-CoV-2 spike protein was retrieved from the UniProt database (accession number P0DTC2). To focus the analysis on the RBD, all regions outside the RBD segment were manually removed, ensuring that only residues corresponding to the RBD were retained for downstream structure prediction. The amino acid sequences of the designed peptides A13 and Nat1, along with the truncated RBD sequence, were then submitted to the AlphaFold 3 web interface for structure prediction and interaction modeling. AlphaFold 3 employs an advanced deep-learning neural network that integrates both sequence and structural information to predict high-confidence protein–protein interactions. Upon completion of the modeling process, the software generated a predicted 3D complex structure illustrating the potential binding interface between each peptide and the RBD.

Following prediction, the resulting Protein Data Bank (PDB) files were imported into PyMOL 3.0 (Schrödinger, LLC, New York, NY, USA) for structural analysis and visualization. Within PyMOL, the molecular surfaces of the peptides and RBD were rendered, and the putative interaction sites were identified using the “select interface” command. A distance cutoff of 5 angstroms (Å) was applied to define interface residues, meaning that any peptide and RBD atoms within 5 Å of each other were considered to be part of the interaction interface. This approach allowed clear visualization of the potential contact regions and provided insight into the molecular determinants of peptide–RBD binding.

### 4.2. Target Binding Analysis

To evaluate the binding of peptides to the RBD region, a direct ELISA was conducted as before [16]. In brief, a mixture that included total human protein (25 ng/mL) with or without recombinant HIS-tagged S1 protein RBD (10 ng/mL) was immobilized onto a HIS antibody-tagged ELISA plate, followed by incubation with FLAG-tagged (DYKDDDDK) A13 and Nat1. Detection was performed using an anti-FLAG monoclonal antibody conjugated to horseradish peroxidase (HRP) and signal development was measured using a 96 well microplate reader. The negative control peptide containing randomly incorporated amino acids was also included. For RBD-ACE2 competition assay, RBD was first immobilized on to the wells followed by different concentrations of the peptides (0, 0.1 and 1 µM) and then incubated with HIS-tagged ACE2 protein. Detection was performed using anti-HIS monoclonal antibodies conjugated with HRP and quantified as relative light units (RLUs).

### 4.3. Interaction Inhibition Analysis Between RBD and Human ACE2

The ability of the peptides to block the interaction between the RBD and ACE2 was assessed using a commercial ACE2 inhibition assay kit (BPS Bioscience, San Diego, CA, United States, CAT# 79931). The pre-coated plate was then blocked by blocking buffer provided in the kit followed by the incubation of the peptides A13 or Nat1. Then HIS-tagged ACE2 protein was introduced into each well and the anti-HIS antibody conjugated to HRP was used to detect ACE2 protein. Finally, the chemiluminescence was detected by plate reader (BioTek Cytation 96-well plate reader, Agilent Technologies, Inc., Santa Clara, CA, USA).

### 4.4. Inhibition Analysis Using a NanoLuc Bioreporter Assay

A *NanoLuc* complementation-based bioreporter assay was used to further assess the inhibitory effects of the peptides on the RBD region of the S1 protein and ACE2 interaction as described in [36]. The bioreporter system utilized split luciferase fragments fused to RBD and ACE2, respectively, generating a luminescent signal upon interaction. The sequence of RBD was fused with the sequence of LgBiT. The fragment was then cloned into the backbone pcDNA3.1. Similarly, the sequence of ACE2 was fused with the sequence of SmBiT, then cloned into the pcDNA3.1. The 293T (ATCC CRL-3216) cells were utilized to express the two recombinant proteins. After obtaining the cell lysis or supernatant containing RBD-LgBiT and SmBiT-ACE2, peptides A13 or Nat1 were added prior to incubation of the two recombinant proteins. The inhibition of binding was quantified by measuring luminescence in Synergy microplate reader (BioTek, Winooski, VT, USA).

### 4.5. Pseudovirus Infectivity Assay Analysis

A lentiviral-based pseudovirus infectivity assay was performed to evaluate the ability of the peptides to inhibit SARS-CoV-2 entry into ACE2-expressing cells as described [39]. The SARS-CoV-2 spike pseudotyped lentivirus was generated as previously described [40]. Cells expressing SmBiT-ACE2 protein were infected by pseudovirus in the presence or absence of the peptides A13 or Nat1, and viral entry was quantified using by luciferase assay using the Bright-Glo Luciferase Assay system following manufacturer’s protocols (Promega, Madison, WI, USA). Cytotoxicity was assessed in parallel to confirm that inhibition was not due to toxic effects of the peptides.

## Data Availability

The original contributions presented in this study are included in the article. Further inquiries can be directed to the corresponding author.

## Abbreviations

The following abbreviations are used in this manuscript:

SARS-CoV-2	Severe acute respiratory syndrome-coronavirus-2
S1	Spike 1 protein
ELISA	Enzyme-linked immunosorbent assay
RBD	Receptor-binding domain
ACE2	Angiotensin-converting enzyme 2
AI	Artificial intelligence
*InSiPS*	In Silico *Protein Synthesizer*
PPI	Protein–protein interaction
HRP	Horseradish peroxidase
RLU	Relative light unit
Å	Angstroms
